# Supplementary material for: Tumour-retained activated CCR7+ dendritic cells are heterogeneous and regulate local anti-tumour cytolytic activity
Source: Nat Commun. 2024 Jan 24;15:682. doi: 10.1038/s41467-024-44787-1 (PMC10808534; doi:10.1038/s41467-024-44787-1)
Supplement: Supplementary file 1 — Supplementary Information [file 41467_2024_44787_MOESM1_ESM.pdf]

Supplementary information

Tumour-retained activated CCR7+ dendritic cells are heterogeneous and regulate local anti-tumour cytolytic activity

Colin YC Lee[1,2], Bethany C Kennedy[3], Nathan Richoz[1], Isaac Dean[3], Zewen K Tuong[1,2], Fabrina Gaspal[3], Zhi Li[3], Claire Willis[3], Tetsuo Hasegawa[1], Sarah K Whiteside[4], David A Posner[1], Gianluca Carlesso[5], Scott A Hammond[5], Simon J Dovedi[6], Rahul Roychoudhuri[4], David R Withers[3#], Menna R Clatworthy[1,2#]

[1] Molecular Immunity Unit, Department of Medicine, Medical Research Council Laboratory of Molecular Biology, University of Cambridge, Cambridge, UK

[2] Cellular Genetics, Wellcome Sanger Institute, Wellcome Genome Campus, Hinxton, Cambridge, UK

[3] Institute of Immunology and Immunotherapy, College of Medical and Dental Sciences, University of Birmingham, Birmingham, UK

[4] Department of Pathology, University of Cambridge, Cambridge, UK

[5] Early Oncology R&D, AstraZeneca, Gaithersburg, USA

[6] Early Oncology R&D, AstraZeneca, Cambridge, UK

[#] **Correspondence:**
Prof. David R Withers, d.withers@bham.ac.uk;
Prof. Menna R Clatworthy, mrc38@medschl.cam.ac.uk

Contents

Supplementary figures 1-12
Supplementary table 1: Antibodies and reagents

Provided separately as Supplementary Data files

Supplementary data 1: Gene sets used
Supplementary data 2: Cluster DEGs in scRNA-seq of tumour DCs

Supplementary Figure 1

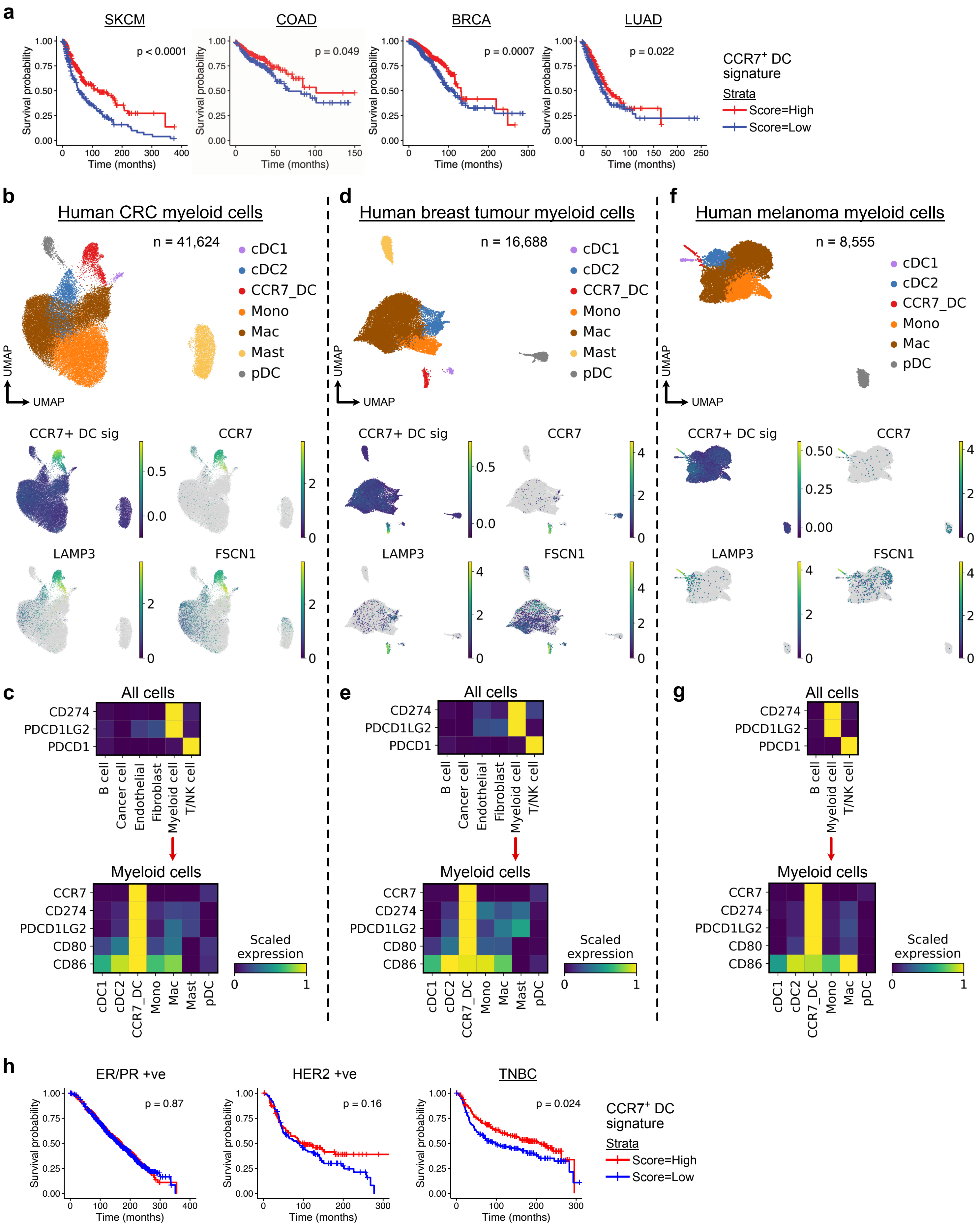

Supplementary Figure 1 | CCR7$^+$ DCs in human cancer.
(**a**) Kaplan-Meier analysis of overall survival rate in skin cutaneous melanoma (SKCM, *n* = 473), colorectal adenocarcinoma (COAD, *n* = 521), breast invasive carcinoma (BRCA, *n* = 1226), and lung adenocarcinoma (LUAD, *n* = 598) from TCGA, stratified by enrichment of CCR7$^+$ DC signature genes. (**b**) Uniform manifold approximation projection (UMAP) of myeloid cells from scRNA-seq of human CRC[26] (*n* = 62 patients) and expression of CCR7$^+$ DC genes (CCR7$^+$ DC signature score[5], *CCR7*, *LAMP3*, *FSCN1*). Mac, macrophage; Mast, mast cell; Mono, monocyte; pDC, plasmacytoid DC. (**c**) Expression of *CD274* (PD-L1), *PDCD1LG2* (PD-L2) and *PDCD1* (PD-1), *CCR7*, *CD80* and *CD86* by cell-type (all cells, top; myeloid cells only, bottom) in scRNA-seq of human CRC. (**d**) UMAP of myeloid cells from scRNA-seq of human breast cancer[24] (*n* = 29 patients) and expression of CCR7$^+$ DC genes. (**e**) Expression of selected genes by cell-type in scRNA-seq of human breast cancer. (**f**) UMAP of myeloid cells from scRNA-seq of human melanoma[25] (*n* = 25 patients) and expression of CCR7$^+$ DC genes. (**g**) Expression of selected genes by cell-type in scRNA-seq of human melanoma. (**h**) Kaplan-Meier analysis of overall survival rate in hormone receptor-positive (oestrogen receptor, ER, or progesterone receptor, PR, *n* = 1369), human epidermal growth factor receptor 2 (HER2)-positive (*n* = 185), or triple-negative breast cancer (TNBC, *n* = 299) from METABRIC cohort. *L*og-rank test for Kaplan-Meier survival was used (**a**, **h**).

Supplementary Figure 2

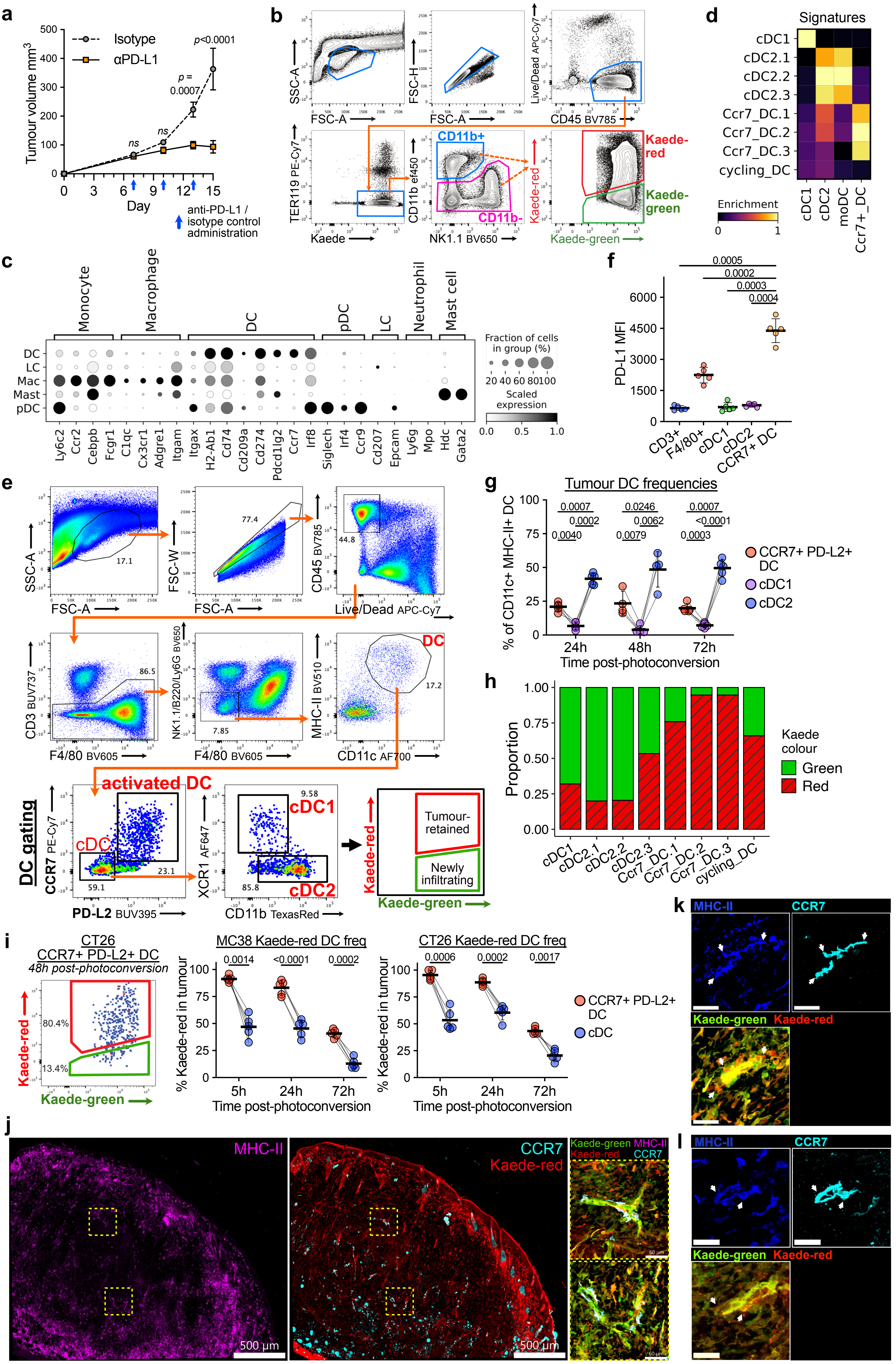

Supplementary Figure 2 | Single cell profiling of mouse subcutaneous tumours.
(**a**) Tumour growth curves of three-dose anti-PD-L1 versus isotype control antibody treatment in MC38-Ova tumours. Data from scRNA-seq experiment (Fig. 1) and includes 5 mice in the isotype control group and 6 mice in the anti-PD-L1 group. (**b**) Fluorescence-activated cell sorting (FACS) strategy for isolation of tumour immune cells for scRNA-seq. $CD45^+$ cells were sorted to $CD11b^+$ or $CD11b^{-/low}$ fractions to ensure appropriate representation of various cell types in the scRNA-seq data. Both fractions were combined for scRNA-seq analysis and annotated based on their transcriptome. (**c**) Canonical marker gene expression in myeloid cell subsets from Fig. 1b. (**d**) Gene signature scores of published transcriptomic reference signatures in DC clusters from Fig. 1c. Ccr7+_DC signature obtained from mRegDC genes in Maier *et al*[5]. Monocyte-derived DCs (moDC) signature scored highly in cDC2 clusters, consistent with known challenges in distinction of cDC2 and moDC[9]. (**e**) Representative flow cytometry gating strategy for tumour and LN DCs (Fig. 1-3). Activated DCs were defined as live, $CD45^+$ $lineage^-$ (CD3, NK1.1, B220, Ly6G, F4/80) $CD11c^+MHC\text{-}II^+$ $PD\text{-}L2^+CCR7^+$ cells (PD-L2 expression was more specific for activated DCs than PD-L1). Where Kaede transgenic mice were used, $Kaede^+$ cells were gated. (**f**) Flow cytometry of surface PD-L1 expression on immune cells from MC38-Ova tumours. (**g**) Flow cytometry of DC composition in MC38-Ova tumours. (**h**) Kaede fluorescence by DC cluster in the scRNA-seq data. (**i**) Flow cytometry of Kaede fluorescence in DCs from MC38 and CT26 tumours; photoconversion time course. (**j**) Representative microscopy of MC38 tumours 72h after tumour photoconversion. Insets highlight selected regions with tumour-residing ($Kaede\text{-}red^+$) $CCR7^+MHC\text{-}II^+$ DCs. (**k-l**) Zoomed-in representative microscopy of MC38 (**k**) and CT26 (**l**) tumours 72h after tumour photoconversion. Arrows point to $Kaede\text{-}red^+CCR7^+MHC\text{-}II^+$ DCs. Scale bar, 30μm. Two-way analysis of variance (ANOVA) and Šidák's multiple comparisons test (**a**), one-way ANOVA and Šidák's multiple comparisons test (**f**), or paired two-sided student's t-test (**g**, **i**) were used. Points represent independent mice (**f**, **g**, **i**). Data are shown as means ± s.e.m. (**a**), or means ± s.d. (**f**, **g**, **i**). The results shown in (**f**) are from one experiment (n = 5 animals), representative of three independent experiments; (**g**) are from one experiment (24h/72h n = 5; 48h n = 4 animals), representative of three independent experiments; (**i**) are from one experiment (n = 5 animals per group), representative of two independent experiments; and (**j-l**) are representative of three independent experiments (n = 7 animals) for MC38 tumours and two independent experiments (n = 4 animals) for CT26 tumours.

Supplementary Figure 3

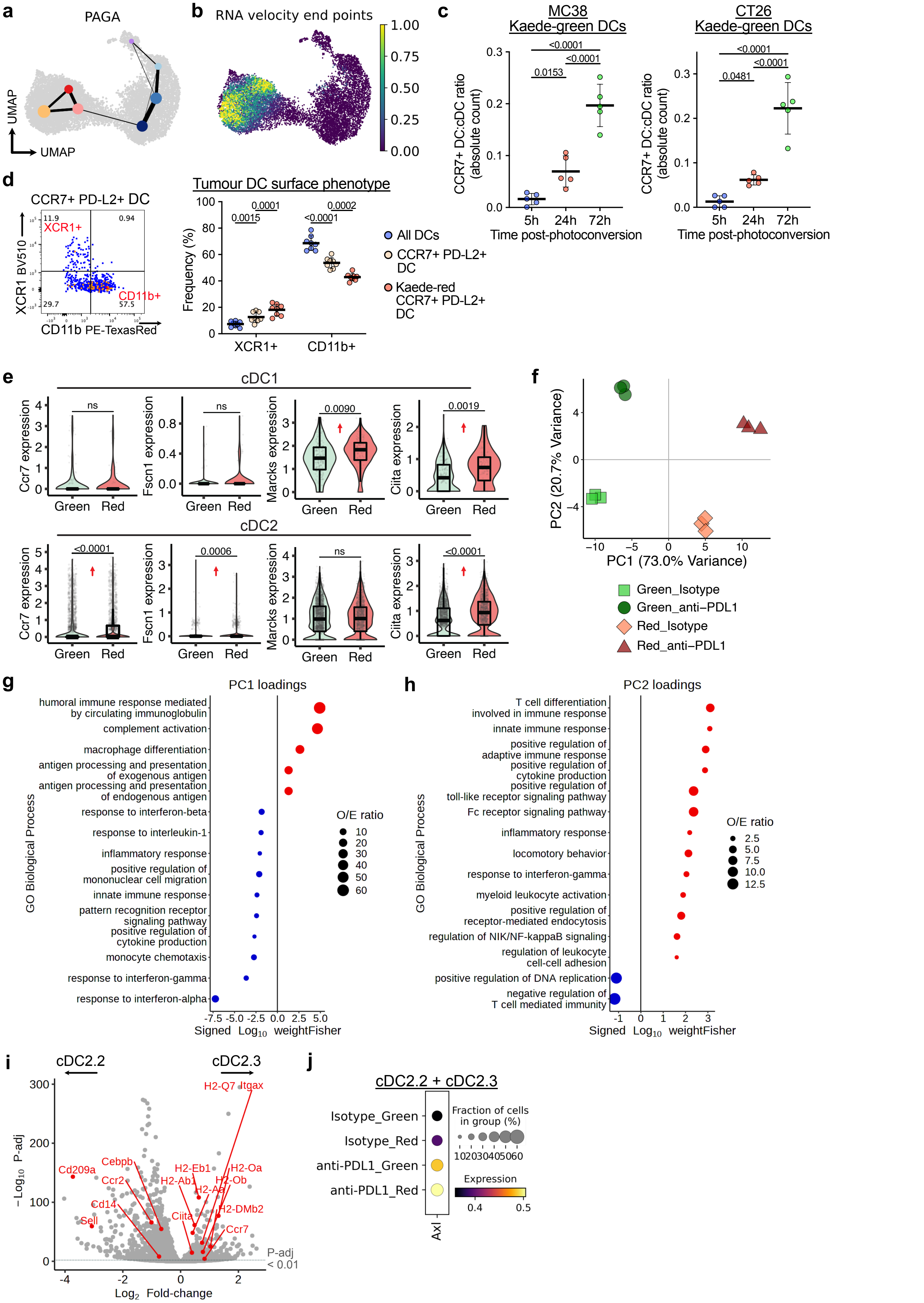

Supplementary Figure 3 | Activation trajectory in tumour DCs.
(**a**) Partition-based graph abstraction (PAGA) of scRNA-seq of tumour DCs. (**b**) Terminal states (end points) of RNA velocity analysis. (**c**) Flow cytometry of CCR7$^+$ DC to cDC ratio in Kaede-green DCs from MC38 and CT26 tumours; time course post-photoconversion. (**d**) Flow cytometry of surface cDC1 (XCR1$^+$) and cDC2 (CD11b$^+$) marker expression on CCR7$^+$ DCs from MC38-Ova tumours 48h post-photoconversion. (**e**) Expression of selected DC migration genes and *Ciita* in scRNA-seq data of cDC1 and cDC2. Arrows indicate relative expression in Kaede-red versus Kaede-green cells.
(**f**) Principal component (PC) analysis of scRNA-seq of cDC2s, pseudo-bulked to 3 artificial replicates per condition. PC1, variance associated with Kaede profile; PC2, variance associated with treatment. (**g-h**) Gene ontology (GO) term enrichment test for the top 100 loading genes of PC1 (**g**) and PC2 (**h**). O/E, observed/expected ratio; signed Fisher weight indicates direction of gene loading. (**i**) Differential gene expression between cDC2.2 and cDC2.3. Selected differentially expressed genes (DEG) relating to DC maturation, antigen presentation, MHC-II and migration are highlighted in red (all P-adj < 0.01). (**j**) Expression of *Axl* in cDC2.2 and cDC2.3 combined. One-way analysis of variance (ANOVA) and Šidák's multiple comparisons test (**c**), paired two-sided student's t-test (**d**), two-sided Wilcoxon rank-sum test with Benjamini-Hochberg multiple-testing correction (**e**), Fisher's exact test (**g-h**), or two-sided Wald test with Benjamini-Hochberg multiple testing correction (**i**) were used. Points represent independent mice (**c-d**). Data are shown as means ± s.d. (**c-d**), or box (median; box, 25th percentile and 75th percentile; whiskers, 1.5*inter-quartile range) and violin plots (**e**). The results shown in (**c**) are from one experiment (n = 5 animals per group), representative of two independent experiments; and (**d**) are from two independent experiments (n = 8 animals).

Supplementary Figure 4

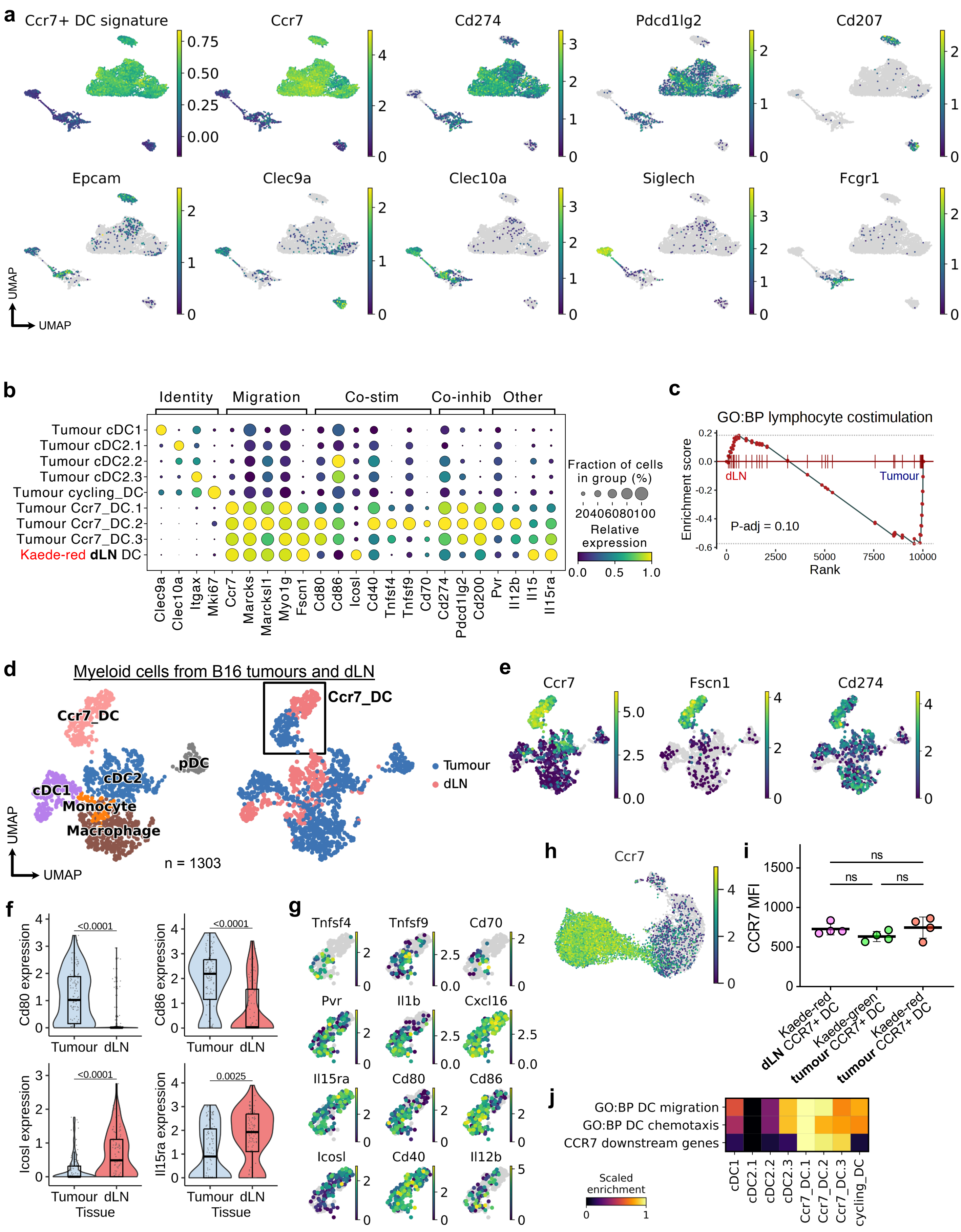

Supplementary Figure 4 | Comparison of tumour-residing CCR7$^+$ DCs versus LN CCR7$^+$ DC emigrants.

(**a**) Expression of CCR7$^+$ DC or myeloid cell canonical marker genes in scRNA-seq of LN myeloid cells (Fig. 2d). (**b**) Expression of selected genes in tumour DCs (isotype control-treated) and Kaede-red DC emigrants in tumour-dLNs. (**c**) GSEA of "*GO:biological process (BP) lymphocyte co-stimulation (GO:0031294)*", comparing Kaede-red CCR7$^+$ DCs from the dLN versus tumour CCR7$^+$ DCs. P-adj, Benjamini-Hochberg-adjusted p values. (**d**) UMAP of myeloid cells from scRNA-seq of murine subcutaneous B16 tumours and tumour-dLNs, by cell-type and tissue. (**e**) Expression of CCR7$^+$ DC genes in (**d**). (**f**) Violin plots of selected genes in scRNAseq of CCR7$^+$ DCs from B16 tumours and tumour-dLNs. (**g**) Expression of selected DEGs between in tumour and dLN CCR7$^+$ DCs from (**d**, box). (**h-i**) Expression of CCR7 in DCs from MC38-Ova tumours in the scRNA-seq data (**h**) and by flow cytometry (**i**), 48h post-photoconversion. (**j**) Gene signature enrichment of pathways relating to DC movement and CCR7 downstream signalling. Two-sided Wilcoxon rank-sum test with Benjamini-Hochberg multiple-testing correction (**f**), or one-way analysis of variance (ANOVA) and Šidák's multiple comparisons test (**i**) were used. Points represent independent mice (**i**). Data are shown as box (median; box, 25th percentile and 75th percentile; whiskers, 1.5*inter-quartile range) and violin plots (**f**), or means ± s.d. (**i**). The results shown in (**i**) are from one experiment (n = 4 animals per group), representative of three independent experiments.

Supplementary Figure 5

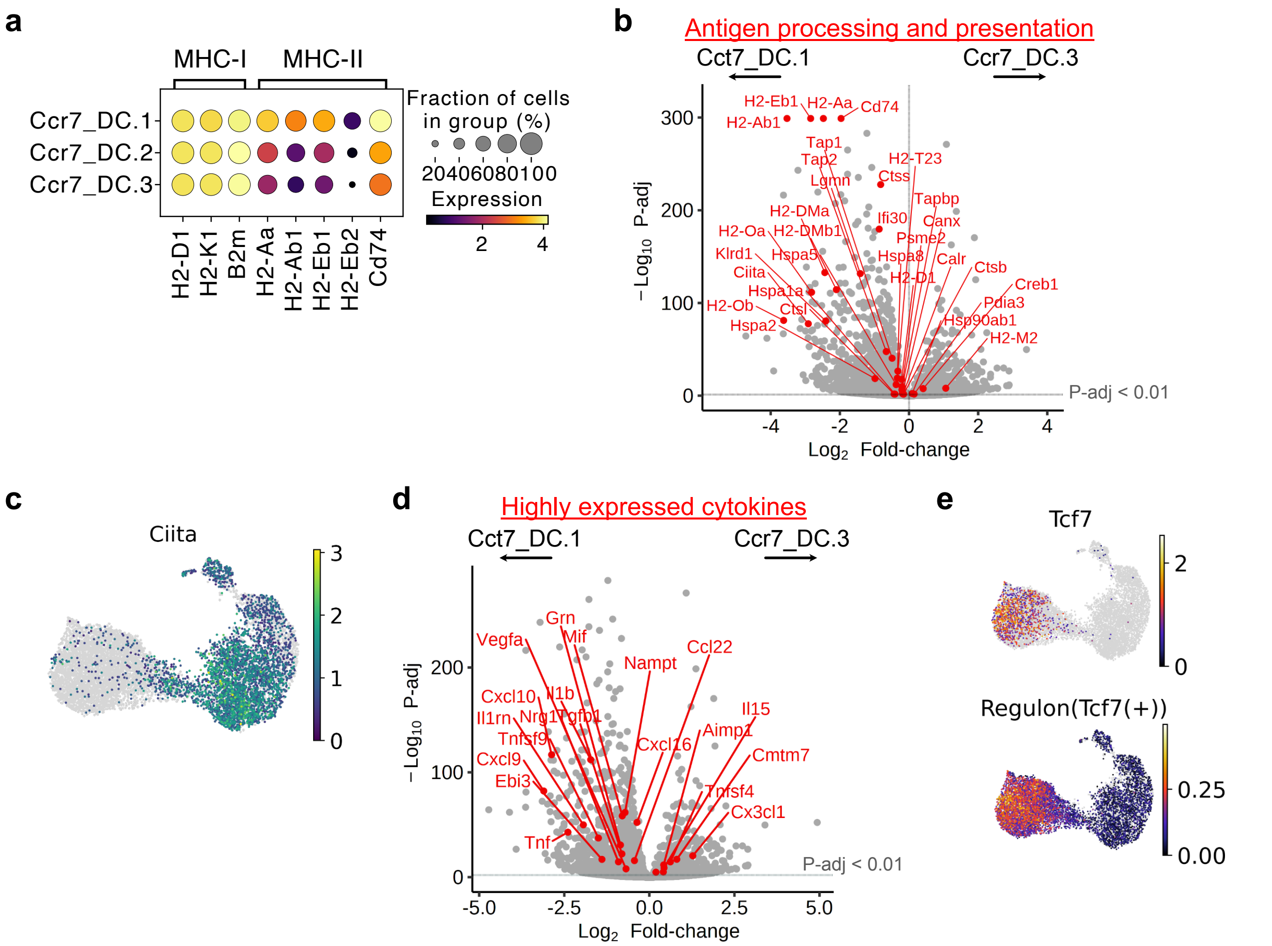

Supplementary Figure 5 | Transcriptional changes in CCR7⁺ DCs with prolonged tumour residence. (**a**) Dot plot of class-I and class-II MHC gene expression (log-transformed, unscaled) in CCR7⁺ DCs. (**b**) Differential gene expression between Ccr7_DC.1 and Ccr7_DC.3. Significant DEGs from "*KEGG antigen processing and presentation*" are highlighted in red (*P*-adj < 0.01). (**c**) Expression of *Ciita*. (**d**) Differential gene expression between Ccr7_DC.1 and Ccr7_DC.3. Significant DEGs from "*GO: molecular function (MF) cytokine activity (GO:0005125)*" are highlighted in red (*P*-adj < 0.01). (**e**) *Tcf7* expression and regulon activity score. Two-sided Wald test with Benjamini-Hochberg multiple testing correction (**b**, **d**) was used.

Supplementary Figure 6

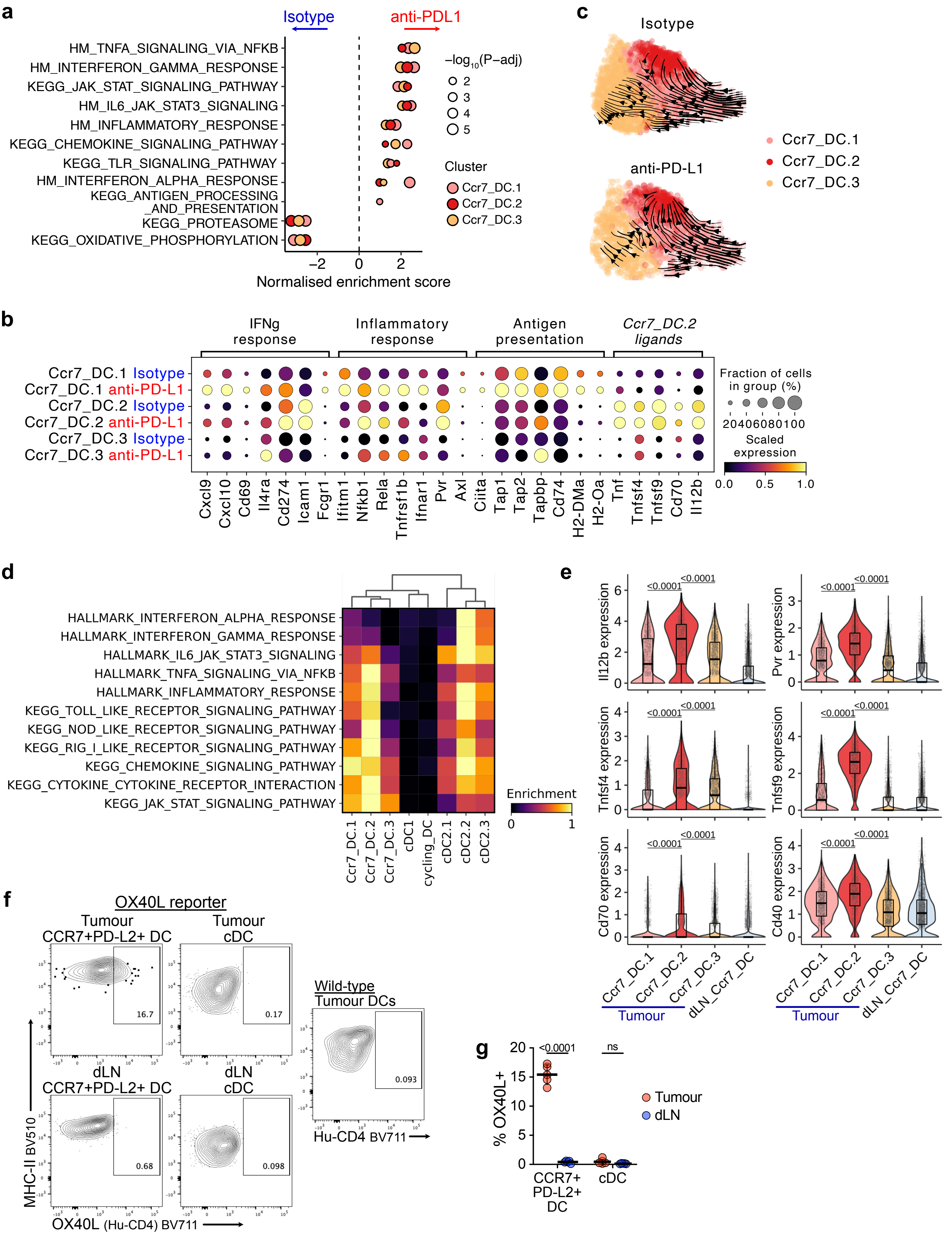

Supplementary Figure 6 | CCR7$^+$ DC activation following anti-PD-L1 treatment.
(**a**) GSEA of CCR7$^+$ DCs in anti-PD-L1-treated versus isotype control-treated tumours; Hallmark (HM) and KEGG pathways. Only significant pathways (Benjamini-Hochberg-adjusted p values (P-adj) < 0.05) are shown. (**b**) Dot plot of selected leading-edge genes from GSEA analysis of "*Hallmark interferon gamma response*", "*Hallmark inflammatory response*", "*KEGG antigen processing and presentation*" pathways from (**a**), and others. (**c**) RNA velocity trajectory in tumour CCR7$^+$ DCs split by treatment group. (**d**) Gene signature enrichment of selected pathways in scRNA-seq of DCs. (**e**) Expression of selected ligands differentially expressed between scRNA-seq of tumour CCR7$^+$ DCs and CCR7$^+$ DC tumour emigrants in the dLN (Kaede-red). (**f**) Representative flow cytometry of surface OX40L expression (using OX40L$^{+/Human\text{-}CD4}$ reporter mice) on DCs from MC38-Ova tumours and dLNs. Wild-type DC (non-reporter) were used to determine OX40L$^+$ gate. (**g**) Quantification of (**f**). Two-sided Wilcoxon rank-sum test with Benjamini-Hochberg multiple-testing correction (**e**), or paired two-sided student's t-test (**g**) were used. Points represent independent mice (**g**). Data are shown as box (median; box, 25th percentile and 75th percentile; whiskers, 1.5*inter-quartile range) and violin plots (**e**), or means ± s.d. (**g**). The results shown in (**f-g**) are from one experiment (n = 5 animals), representative of two independent experiments.

Supplementary Figure 7

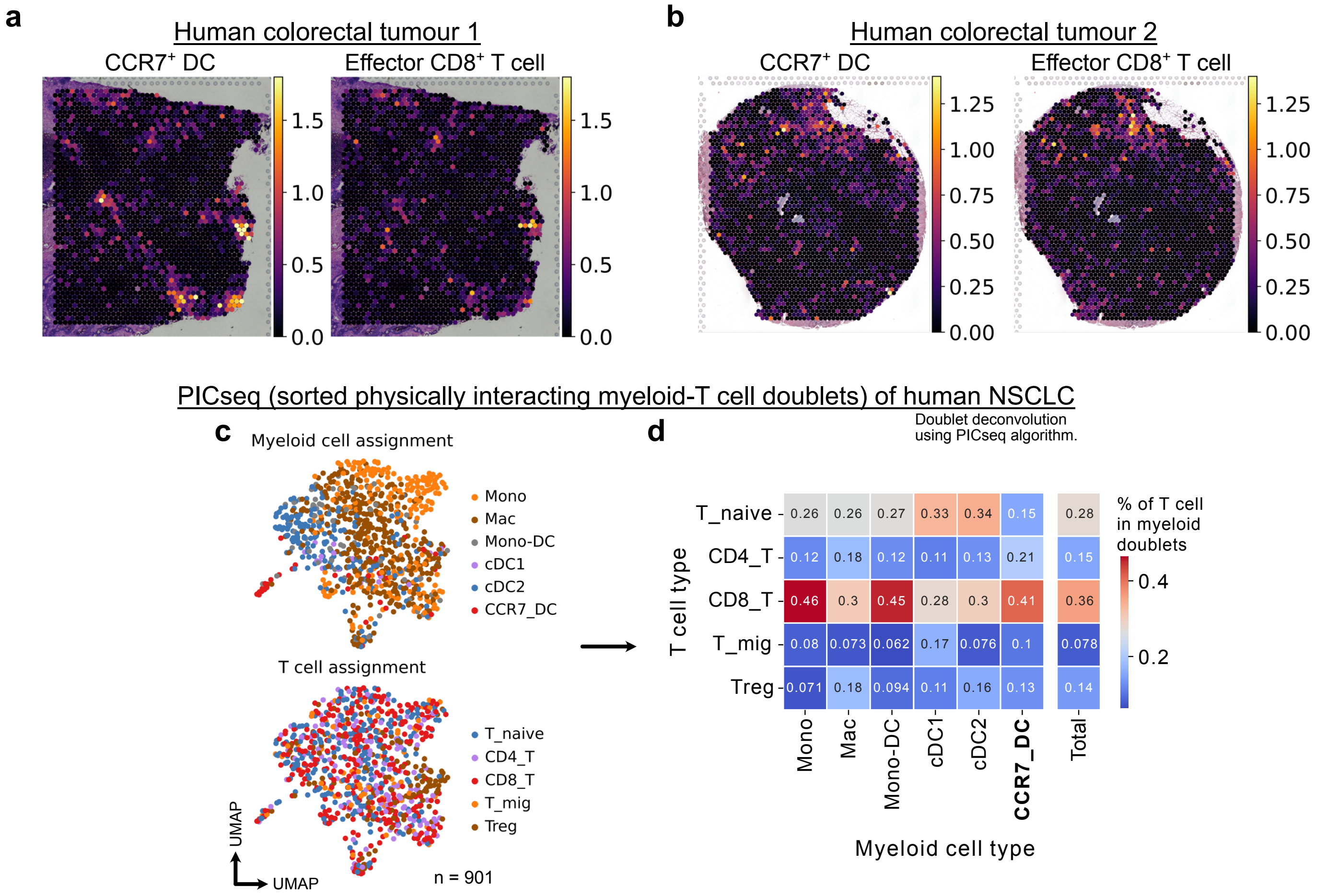

Supplementary Figure 7 | CCR7+ DC-CD8+ T cell interaction in human solid tumours. (**a-b**) Gene signature scores for CCR7+ DCs and effector CD8+ T cells in spatial transcriptomics (10X Genomics Visium) of independent human CRC tumour sections associated with Fig. 4d (*n* = 2). (**c**) Sequencing of physically interacting cells (PICseq, myeloid-T cell doublets) from human NSCLC (*n* = 10 patients). UMAP of PICs coloured by the deconvolved myeloid and T cell assignment for each doublet (PICseq algorithm). Each dot represents one myeloid-T cell doublet. (**d**) Heatmap of frequency (values) of myeloid-T cell doublet combinations in PICseq of NSCLC.

Supplementary Figure 8

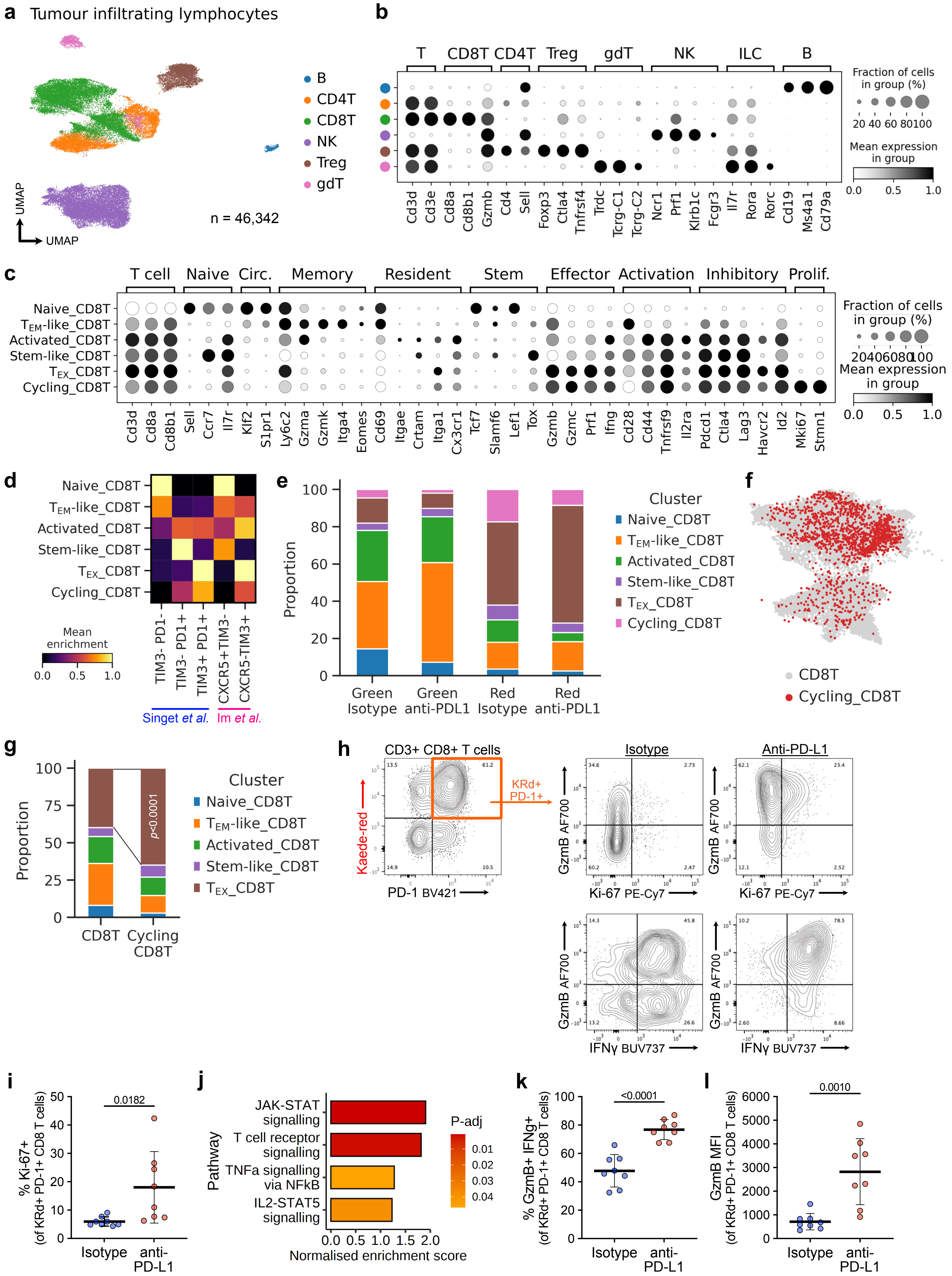

Supplementary Figure 8 | Single cell profiling of tumour $CD8^+$ T cells following anti-PD-L1 treatment.
(**a**) UMAP of TILs from scRNA-seq of FACS-sorted $CD45^+TER119^-$ Kaede-green$^+$/Kaede-red$^+$ cells 48h after photoconversion of subcutaneous MC38-Ova tumours, and canonical marker gene expression (**b**). (**c**) Canonical marker gene expression of $CD8^+$ T cell clusters from Fig. 5a. (**d**) Gene signature enrichment of published T cell exhaustion transcriptomic signatures in $CD8^+$ T cell clusters. (**e**) Proportion of $CD8^+$ T cells by Kaede fluorescence and treatment group. (**f**) Regression of cell cycle genes from the cycling_CD8T cluster followed by re-integration, and label transfer. Majority of the cycling cluster embedded with the $CD8^+$ T_{EX} cell cluster. (**g**) Proportion of cycling_CD8T cells belonging to each $CD8^+$ T cell cluster, from (**f**). (**h**) Representative flow cytometry of Ki-67, GzmB and IFNγ expression in $CD3^+CD8^+$Kaede-red$^+$PD-1$^+$ T cells; isotype control and anti-PD-L1 treatment; MC38-Ova tumours (**h-l**). (**i**) Flow cytometry of Kaede-red$^+$PD-1$^+$CD8$^+$ T cells, showing increased frequency of cycling cells (Ki-67$^+$) following anti-PD-L1. (**j**) GSEA for T_{EX} $CD8^+$ T cells from anti-PD-L1 versus isotype control-treated tumours. P-adj, Benjamini-Hochberg-adjusted p values. (**k**) Flow cytometry of Kaede-red$^+$PD-1$^+$CD8$^+$ T cells, showing increased frequency of GzmB$^+$IFNγ$^+$ cells following anti-PD-L1. (**l**) Flow cytometry of Kaede-red$^+$PD-1$^+$ $CD8^+$ T cells, showing increased expression (MFI) of GzmB following anti-PD-L1. Chi-squared test for over-representation of T_{EX} $CD8^+$ T cells (**g**), or two-sided student's t-test (**i**, **k**, **l**) were used. Points represent independent mice and data are shown as means ± s.d. (**i**, **k**, **l**). The results shown in (**h-i**, **k-l**) are from one experiment (n = 8 animals), representative of two independent experiments.

Supplementary Figure 9

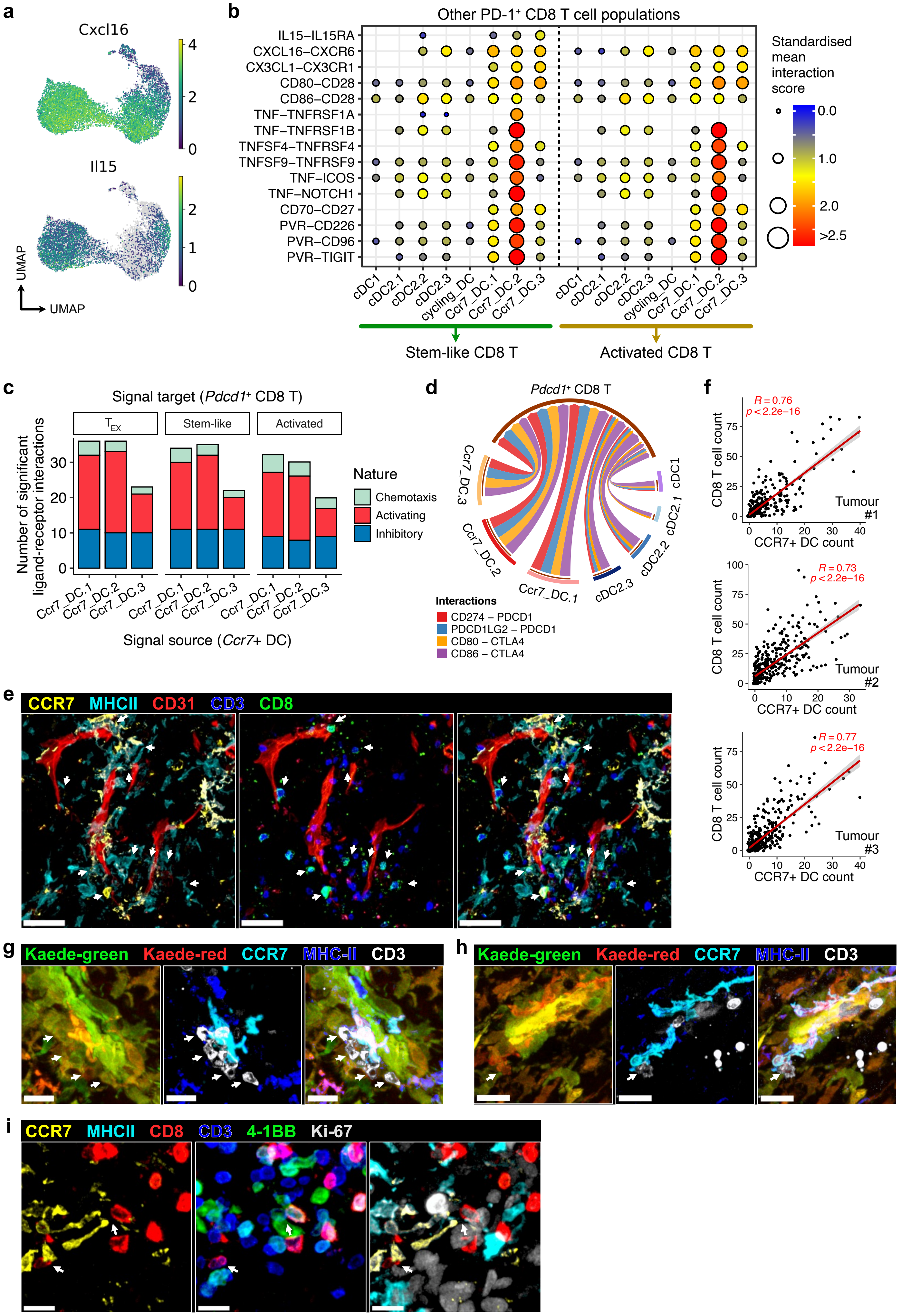

Supplementary Figure 9 | CCR7$^+$ DC-CD8$^+$ T cell interaction in murine tumours.
(**a**) Expression of *Cxcl16* and *Il15*. (**b-d**) CellPhoneDB cell-cell communication analysis between DCs and *Pdcd1*$^+$ CD8$^+$ T cells in scRNA-seq of MC38-Ova tumours. (**b**) Ligand-receptor predicted interactions between tumour DCs and activated or stem-like CD8$^+$cells. (**c**) Interactions that are well-described in existing literature to influence CD8$^+$ T cells function were identified and classified based on whether engagement of the cognate T cell receptor was activating (increase in effector function, proliferation, or survival) or inhibitory in nature. (**d**) *PDCD1* or *CTLA4*-mediated inhibitory signals; edge width scaled to standardised interaction scores. Only significant interactions ($p < 0.05$) shown (**b-d**). (**e**) Representative confocal microscopy images of MC38 tumours, showing co-localisation of CCR7$^+$MHC-II$^+$ DCs and CD3$^+$CD8$^+$ T cells (arrows). Scale bar, 80 µm. (**f**) Quantification of (**e**); Pearson correlation between CCR7$^+$ DC and CD8$^+$ T cell counts in 200 x 200 µm fields of MC38 tumour sections. 3 independent tumours were analysed. (**g-h**) Representative microscopy of independent MC38 tumours 48h after tumour photoconversion, showing interactions between Kaede-red CCR7$^+$MHC-II$^+$ DCs and Kaede-red CD3$^+$ T cells (arrows). Scale bar, 15 µm (**g**), 20 µm (**h**). (**i**) Representative microscopy of independent MC38 tumour, showing co-localisation of CCR7$^+$MHC-II$^+$ DCs and CD3$^+$CD8$^+$ 4-1BB$^+$/Ki-67$^+$ T cells (arrows). Scale bar, 15 µm. Two-sided Pearson correlation was used (**f**). Data are shown as linear regression with 95% confidence interval (**f**). The results shown in (**e**) are representative of two independent experiments (n = 3 animals); and (**g-i**) are representative of two independent experiments (n = 5 animals).

Supplementary Figure 10

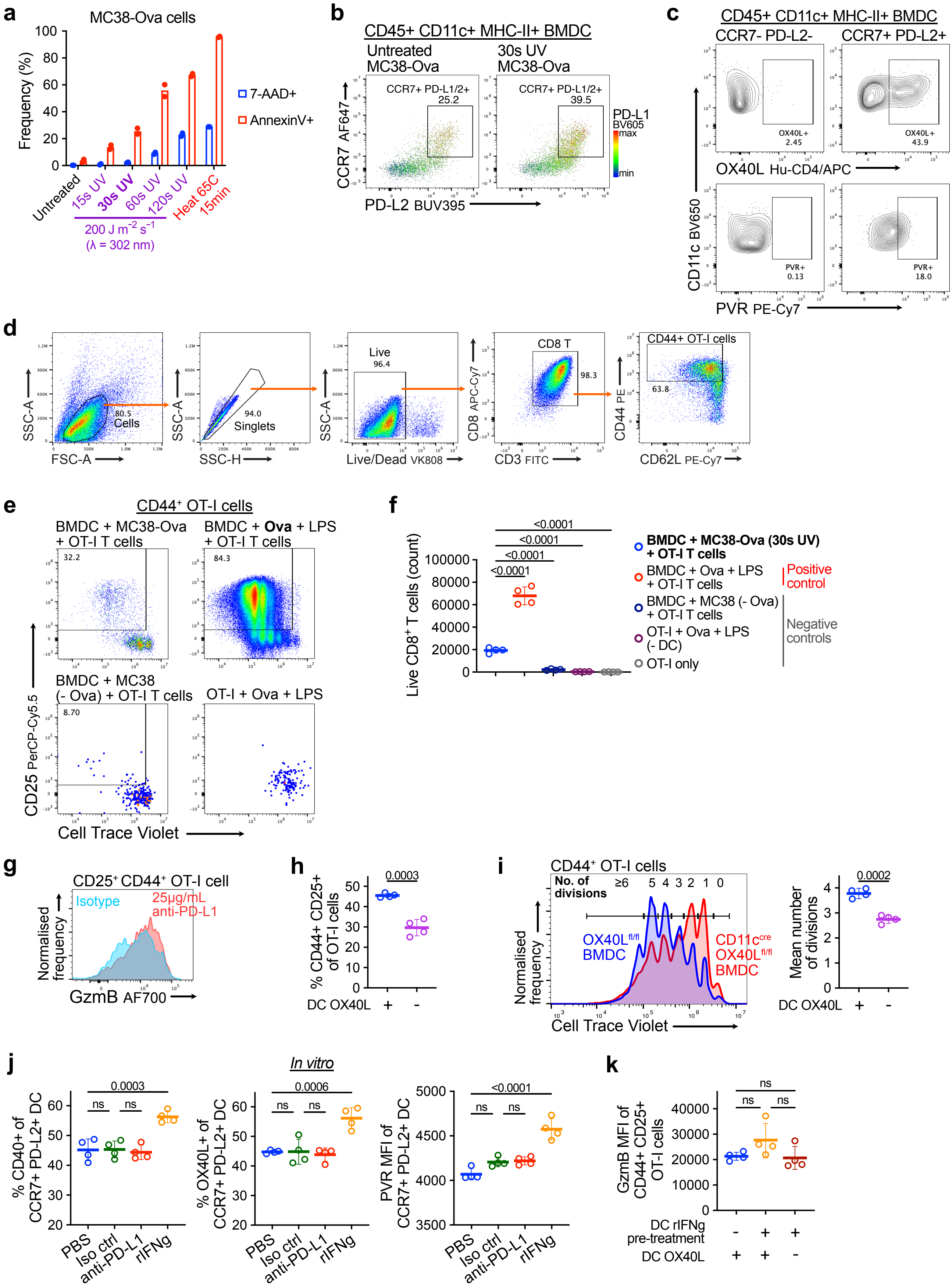

Supplementary Figure 10 | *In vitro* DC and OT-I CD8$^+$ T cell cultures.
(**a**) Apoptosis and cell death following UV irradiation of MC38-Ova cell monolayer *in vitro*; for optimisation of tumour cell-line apoptosis. 30s UV exposure was used for subsequent experiments. (**b**) Representative flow cytometry and FACS isolation strategy for activated BMDC following 8h culture with UV-irradiated MC38-Ova cells (Fig. 5f-g). (**c**) Representative flow cytometry of OX40L and PVR expression on BMDCs. (**d**) Flow cytometry gating strategy for CD44$^+$ OT-I T cells (Fig. 5g-k). (**e**) Representative flow cytometry of OT-I activation and proliferation in DC co-cultures. Culture with apoptotic MC38-Ova experienced FACS-sorted CCR7$^+$PD-L2$^+$ BMDC, top left; positive control (BMDC + Ova + LPS), top right; negative controls, bottom left (no Ova antigen) and right (no DC). (**f**) Number of live CD8$^+$ T cells in CCR7$^+$PD-L2$^+$ BMDC + OT-I co-culture set-up versus controls. (**g**) Representative flow cytometry histograms of GzmB expression in OT-I cells; +/- anti-PD-L1 antibodies. (**h**) Flow cytometry of OT-I activation, and (**i**) CTV proliferation in OT-I cells and mean number of cell divisions, following culture with OX40L-expressing (+, OX40L$^{fl/fl}$) or OX40L-deficient (-, CD11c^{cre}OX40L$^{fl/fl}$) CCR7$^+$PD-L2$^+$ BMDC. (**j**) Flow cytometry of CCR7$^+$PD-L2$^+$ BMDCs following 8h treatment with antibodies or recombinant IFNγ (rIFNg), which were used for OT-I co-cultures in subsequent independent experiments. (**k**) Flow cytometry of OT-I cells following co-culture with CCR7$^+$PD-L2$^+$ BMDC; DCs pre-treated with rIFNg (+) or PBS (-), OX40L-expressing (+) or OX40L-deficient (-) BMDCs. One-way analysis of variance (ANOVA) and Šidák's multiple comparisons test (**f**, **j-k**), or two-sided student's t-test (**h-i**) was used. Points represent independent biological samples (**a**, **f**, **h-k**). Data are shown as means (**a**), or means ± s.d. (**f**, **h-k**). The results shown in (**a**) is from one experiment; (**b-c**) are from one experiment (*n* = 4 biological samples), representative of three independent experiments; and (**d-k**) are from one experiment (*n* = 4 biological samples), representative of two independent experiments.

Supplementary Figure 11

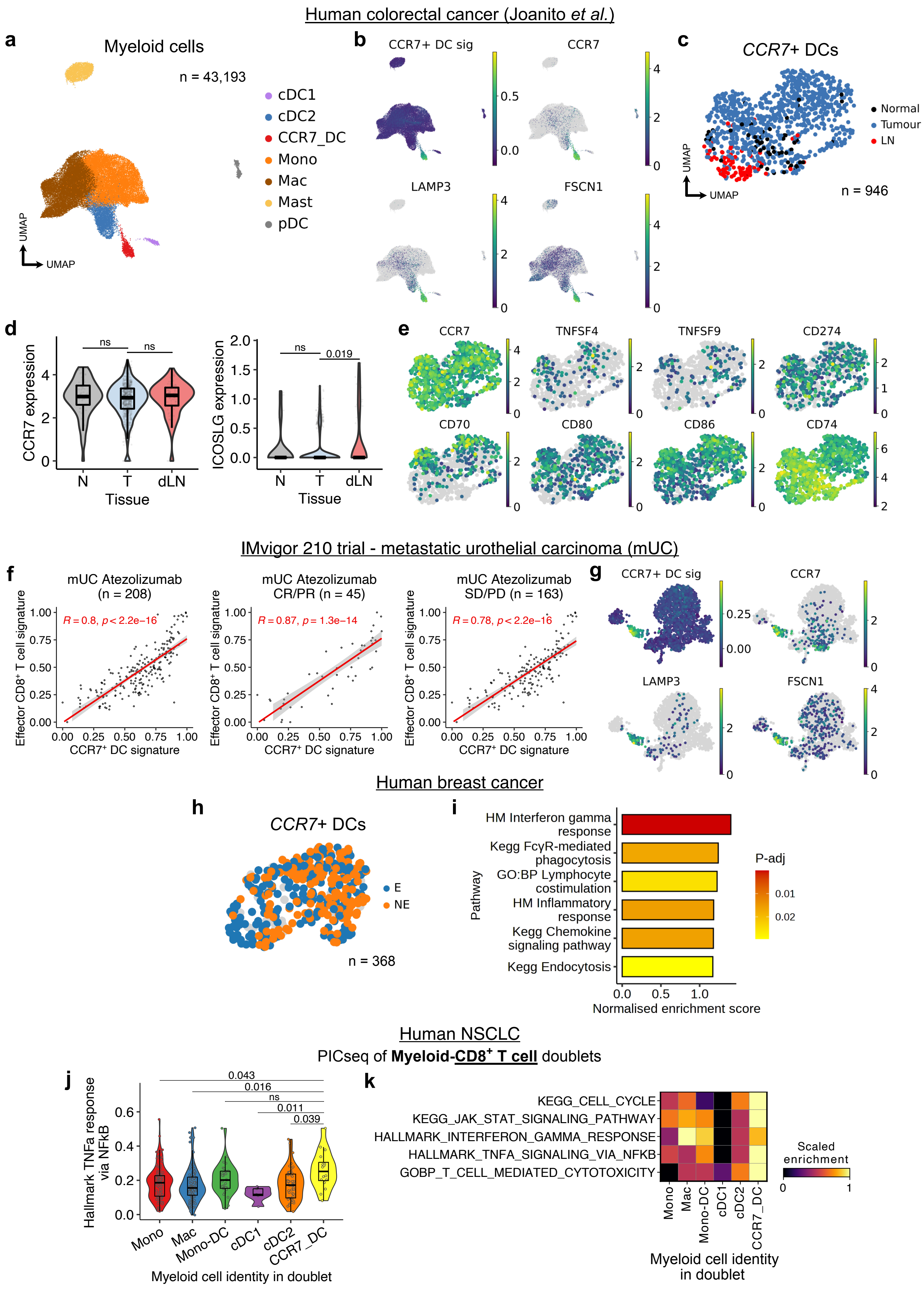

Supplementary Figure 11 | CCR7$^+$ DC heterogeneity and ligand-receptor interactions with CD8$^+$ T cells in human tumours.

(**a**) UMAP of myeloid cells from scRNA-seq of human CRC[42] (*n* = 63 patients) with paired tumour (T), normal adjacent tissue (N) and dLN samples, and expression of CCR7$^+$ DC signature genes (**b**). (**c**) UMAP of scRNA-seq of CCR7$^+$ DCs from (**a**), coloured by tissue cells were retrieved from. (**d**) Expression of *CCR7* and *ICOSLG* in CCR7$^+$ DCs by tissue. (**e**) Expression of selected genes in CCR7$^+$ DCs from (**c**). Molecules associated with tumour-residing CCR7$^+$ DCs in mice were also preferentially expressed in tumour CCR7$^+$ DCs in human CRC, but not the dLN. (**f**) Pearson correlation between CCR7$^+$ DC signature genes and effector CD8$^+$ T cell signature genes in bulk RNA-seq of 208 mUC tumours treated with atezolizumab (IMvigor 210 trial[44]). Left, middle and right panel show all patients, clinical responders, and non-responders respectively. Points represent individual patient samples. (**g**) Expression of CCR7$^+$ DC signature genes in myeloid cells from scRNA-seq of human mUC[46] (*n* = 11 patients). (**h**) UMAP of scRNA-seq of CCR7$^+$ DCs from human breast cancer[24] (T cell clonotype expanders (E), i.e. responders, *n* = 9 patients; non-expanders (NE), i.e. non-responders, *n* = 20 patients; total *n* = 29 patients). (**i**) GSEA of CCR7$^+$ DCs from responders versus non-responders, in breast tumours treated with anti-PD-1 antibodies. P-adj, Benjamini-Hochberg-adjusted p values. (**j**) Gene signature scores for "*Hallmark TNFa response via NFkB*" in PICseq of NSCLC[38], grouped by the myeloid cell identity in each myeloid-CD8$^+$ T cell doublet. (**k**) Gene signature scores of selected pathways in myeloid-CD8$^+$ T cell physically interacting doublets, grouped by the myeloid cell identity. Two-sided Wilcoxon rank-sum test with Benjamini-Hochberg multiple-testing correction (**d**), two-sided Pearson correlation (**f**), or two-sided Wilcoxon rank-sum test (**j**) was used. Data are shown as box (median; box, 25th percentile and 75th percentile; whiskers, 1.5*inter-quartile range) and violin plots (**d**, **j**), or linear regression with 95% confidence interval (**f**).

Supplementary Figure 12

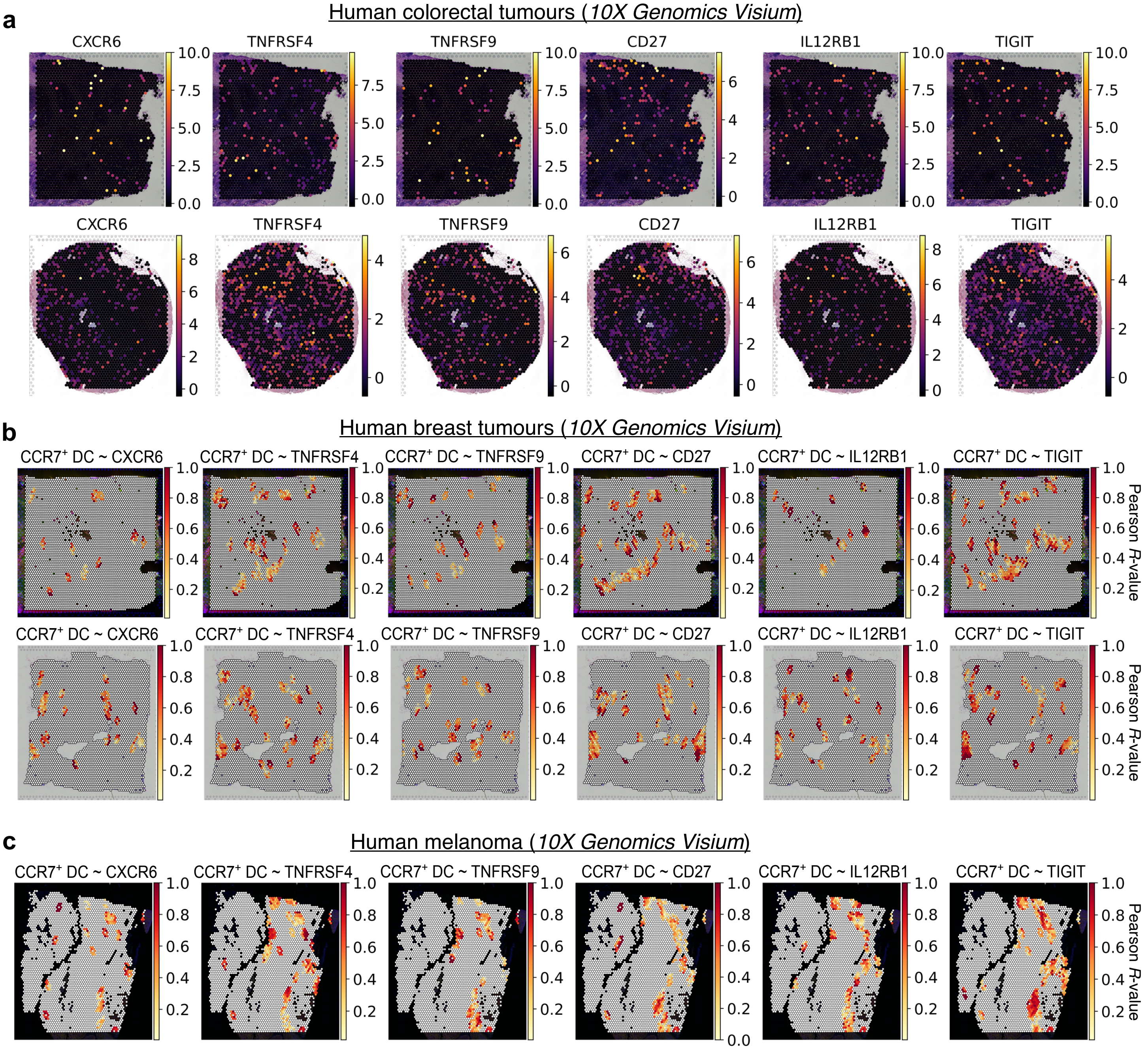

Supplementary Figure 12 | Visium spatial transcriptomics of human cancer.
(**a**) Expression of selected receptors expressed by $CD8^+$ T cells which facilitate interactions with tumour-residing $CCR7^+$ DCs in spatial transcriptomics (10X Genomics Visium) of independent human CRC tumour sections ($n = 2$). (**b-c**) Spatial correlation (Pearson R-value) of $CCR7^+$ DC signature scores and selected $CCR7^+$ DC-ligand receptors expressed by $CD8^+$ T cells, in spatial transcriptomics of independent human breast tumour sections (**b**, $n = 2$) and a human melanoma section (**c**, $n = 1$).

Supplementary table 1: Antibodies and reagents

Target	Fluorophore	Clone	Supplier	Catalogue no.	Dilution	Application
CCR7	AF647	EPR23192-57	Abcam	ab275165	1/100	Microscopy
MHC Class II I-A/I-E	Pacific Blue	M5/114.15.2	BioLegend	107620	1/50	Microscopy
CD3	AF488 / AF647	17A2	BioLegend	100209 / 100210	1/100	Microscopy
CD8a	PE	53-6.7	BioLegend	100707	1/50	Microscopy
CD31	AF594	MEC13.3	BioLegend	102520	1/100	Microscopy
4-1BB	-	AF6-120.1	R&D Systems	AF937	1/200	Microscopy
Ki-67	PE	SolA15	ThermoFisher	12-5698-82	1/50	Microscopy
Donkey anti-goat	AF647	Polyclonal	ThermoFisher	A32849	1/200	Microscopy
CD16/32	-	2.4G2	BioLegend	101302	1/100	Flow
LIVE/DEAD™ Viability dye	APC-Cy7 / NIR	-	ThermoFisher	L10119 / L34981	1/500	Flow
Viability dye	VK808	-	Beckman Coulter	C36628	1/250	Flow
CellTrace Violet	-	-	ThermoFisher	C34571	1/1000	Flow
CD45	BV785 / BUV395	30-F11	BioLegend / BD	103149 / 564279	1/200	Flow
CD11c	AF647 / AF700 / BV650	N418	Invitrogen	56-0114-82 / 416-0114-82	1/200	Flow
CD11b	PE-594 / BV785 / FITC	M1/70	BioLegend	101255 / 101243 / 101205	1/200	Flow
XCR1	AF647 / BV510	ZET	BioLegend	148213 / 148218	1/200	Flow
Ly6C	BV421 / BV711	HK1.4	BioLegend / BD	128031 / 755195	1/250	Flow
MHC Class II I-A/I-E	Pacific Blue / BV510	M5/114.15.2	BioLegend	107620 / 107636	1/200	Flow
CCR7	PE-Cy7 / PE	4B12	Invitrogen	12-1971-82	1/100	Flow
PD-L1	BV711 / BV605	10F.9G2	BioLegend	124319 / 124321	1/100	Flow
PD-L2/CD273	BUV395	TY25	BD Horizon	565102	1/200	Flow
CD40	FITC	HM40-3	ThermoFisher	11-0402-82	1/100	Flow
CD155 (PVR)	PE-Cy7	TX56	BioLegend	131511	1/100	Flow
F4/80	BV605	BM8	BioLegend	123133	1/100	Flow
Ly6G	BV650	1A8	BioLegend	127641	1/200	Flow
CD3	BV650 / FITC	17A2	BioLegend	100229 / 100203	1/100	Flow
CD3e	FITC / BV605	145-2C11	BD / BioLegend	100305 / 100351	1/100	Flow
CD8a	BV510 / BV711	53-6.7	BioLegend	100751 / 100747	1/200	Flow
CD8b	APC-Cy7	YTS156.7.7	BioLegend	126619	1/200	Flow
B220	BV650	RA3-6B2	BioLegend	103241	1/200	Flow
NK1.1	BV650	PK136	BD	564143	1/200	Flow
PD-1	BV421 / APC / BV605	29F.1A12	BioLegend	135217 / 135209 / 135219	1/250	Flow
Ki-67	PE-Cy7	SolA15	eBioscience	25-5698-82	1/200	Flow
Granzyme B	AF700	QA16A02	BioLegend	372222	1/200	Flow
IFNγ	BUV737	XMG1.2	BD	612769	1/200	Flow
CD62L	PE-Cy7	MEL-14	BioLegend	104418	1/200	Flow
CD44	PE	IM7	ThermoFisher	12-0441-82	1/400	Flow
CD25	PerCP-Cy5.5	PC61.5	Invitrogen	45-0251-82	1/200	Flow
Human CD4	APC	RPA-T4	BioLegend	300514	1/100	Flow
Human CD4	BV711	OKT4	BioLegend	317439	1/100	Flow
CD45	BV785 / BUV395	30-F11	BioLegend	103149 / 564279	1/200	FACS
CD11b	eFluor 450 / BV785	M1/70	Invitrogen / BioLegend	48-0112-82 / 101243	1/200	FACS
Ter119	PE-Cy7	TER-119	Invitrogen	25-5921-82	1/250	FACS
LIVE/DEAD™ Viability dye	APC-Cy7	-	ThermoFisher	L10119	1/500	FACS
NK1.1	BV650	PK136	BD	564143	1/200	FACS
B220	BV421	RA3-6B2	BioLegend	103239	1/200	FACS
CD11c	AF700 / PE	N418	Invitrogen	56-0114-82 / 12-0114-82	1/200	FACS
CD3e	FITC	145-2C11	BioLegend	100305	1/100	FACS
CD8b	APC-Cy7	YTS156.7.7	BioLegend	126619	1/200	FACS
CD62L	PE-Cy7	MEL-14	BioLegend	104418	1/200	FACS
CD44	PE	IM7	ThermoFisher	12-0441-82	1/400	FACS
MHC Class II I-A/I-E	Pacific Blue / BV510	M5/114.15.2	BioLegend	107620 / 107636	1/200	FACS
PD-L2/CD273	BUV395	TY25	BD Horizon	565102	1/200	FACS
