# Supplementary material for: Tumour-retained activated CCR7+ dendritic cells are heterogeneous and regulate local anti-tumour cytolytic activity
Source: Nat Commun. 2024 Jan 24;15:682. doi: 10.1038/s41467-024-44787-1 (PMC10808534; doi:10.1038/s41467-024-44787-1)
Supplement: Supplementary file 3 — Description of Additional Supplementary Files [file 41467_2024_44787_MOESM3_ESM.pdf]

Description of Additional Supplementary Files

Supplementary Data 1
Description: Gene sets used

Supplementary Data 2
Description: Cluster DEGs in scRNA-seq of tumour DCs
