# Supplementary material for: Tumour-retained activated CCR7+ dendritic cells are heterogeneous and regulate local anti-tumour cytolytic activity
Source: Nat Commun. 2024 Jan 24;15:682. doi: 10.1038/s41467-024-44787-1 (PMC10808534; doi:10.1038/s41467-024-44787-1)
Supplement: Supplementary file 6 — Reporting Summary [file 41467_2024_44787_MOESM6_ESM.pdf]

Corresponding author(s): Prof. Menna R Clatworthy
Prof. David R Withers

Last updated by author(s): Dec 6, 2023

Reporting Summary

Nature Portfolio wishes to improve the reproducibility of the work that we publish. This form provides structure for consistency and transparency in reporting. For further information on Nature Portfolio policies, see our Editorial Policies and the Editorial Policy Checklist.

Statistics

For all statistical analyses, confirm that the following items are present in the figure legend, table legend, main text, or Methods section.

n/a	Confirmed	
[ ]	[x]	The exact sample size (*n*) for each experimental group/condition, given as a discrete number and unit of measurement
[ ]	[x]	A statement on whether measurements were taken from distinct samples or whether the same sample was measured repeatedly
[ ]	[x]	The statistical test(s) used AND whether they are one- or two-sided<br>*Only common tests should be described solely by name; describe more complex techniques in the Methods section.*
[x]	[ ]	A description of all covariates tested
[ ]	[x]	A description of any assumptions or corrections, such as tests of normality and adjustment for multiple comparisons
[ ]	[x]	A full description of the statistical parameters including central tendency (e.g. means) or other basic estimates (e.g. regression coefficient) AND variation (e.g. standard deviation) or associated estimates of uncertainty (e.g. confidence intervals)
[ ]	[x]	For null hypothesis testing, the test statistic (e.g. *F*, *t*, *r*) with confidence intervals, effect sizes, degrees of freedom and *P* value noted<br>*Give P values as exact values whenever suitable.*
[x]	[ ]	For Bayesian analysis, information on the choice of priors and Markov chain Monte Carlo settings
[x]	[ ]	For hierarchical and complex designs, identification of the appropriate level for tests and full reporting of outcomes
[ ]	[x]	Estimates of effect sizes (e.g. Cohen's *d*, Pearson's *r*), indicating how they were calculated

Our web collection on statistics for biologists contains articles on many of the points above.

Software and code

Policy information about availability of computer code

Data collection	Flow cytometry data was collected on the LSR Fortessa X-20 (BD) using FACSDiva v8.0.2 software (BD) or CytoFLEX (Beckman Coulter) using CytExpert v2.5 (Beckman Coulter). Confocal microscopy images were acquired using a TCS SP8 (Leica microsystems, Milton Keynes, UK) confocal microscope.
Data analysis	Flow cytometry: FlowJo v10.8.1 (BD).<br>Imaging: Imaris v9.7.2 (Bitplane); QuPath v0.3.2; R v4.0.4<br>Statistics: GraphPad Prism (v10.0.3); R v4.0.4<br>Computational analysis (RNA sequencing):<br>Python v3.8.12<br>R v4.0.4<br>bcl2fastq v2.20.0.422<br>CellRanger v6.1.2<br>Scanpy v1.8.2 (scRNA-seq), v1.9.3 (Visium)<br>Scrublet v0.2.1<br>UMAP v0.5.1<br>msigdbr v7.5.1<br>pyScenic v0.12<br>Palantir v1.0.0<br>velocyto v0.17.15<br>scVelo v0.2.5

Milo v0.1.0
CellPhoneDB v2.1.7
DESeq2 v1.34
fgsea v1.24
topGO v2.50
bbknn v1.5.1
harmonypy v0.0.5
MetaCell v0.3.41
ssGSEA v10.1.0
xCell
Scissor v2.1.0
SpaceRanger v1.2.0
ktplots (github.com/sktuong/ktplots)
pseudobulk (github.com/colin-leeyc/CLpseudobulk)

For manuscripts utilizing custom algorithms or software that are central to the research but not yet described in published literature, software must be made available to editors and reviewers. We strongly encourage code deposition in a community repository (e.g. GitHub). See the Nature Portfolio guidelines for submitting code & software for further information.

Data

Policy information about availability of data

All manuscripts must include a data availability statement. This statement should provide the following information, where applicable:

- Accession codes, unique identifiers, or web links for publicly available datasets
- A description of any restrictions on data availability
- For clinical datasets or third party data, please ensure that the statement adheres to our policy

The scRNA-seq data generated in this study have been deposited on the GEO repository under accession numbers GSE221513 [https://www.ncbi.nlm.nih.gov/geo/query/acc.cgi?acc=GSE221513] and GSE221064 [https://www.ncbi.nlm.nih.gov/geo/query/acc.cgi?acc=GSE221064]. Source data are provided with this paper.

Previously published sequencing data: Published data was accessed and downloaded from public GEO, SRA, EGA and Synapse repositories using the following ascension numbers: scRNA-seq of CRC (GSE178341 and syn26844071); scRNA-seq of breast cancer (EGAS00001004809); scRNA-seq of melanoma (GSE123139); scRNA-seq of mUC (HRA000212); scRNA-seq and PICseq of NSCLC (GSE160903); TCGA (https://portal.gdc.cancer.gov, via TCGAbiolinks); METABRIC; IMvigor210 bulk RNA-seq (EGAS00001004343); 10x Visium (https://www.10xgenomics.com/resources/datasets).

Research involving human participants, their data, or biological material

Policy information about studies with human participants or human data. See also policy information about sex, gender (identity/presentation), and sexual orientation and race, ethnicity and racism.

Reporting on sex and gender	n/a
Reporting on race, ethnicity, or other socially relevant groupings	n/a
Population characteristics	n/a
Recruitment	n/a
Ethics oversight	n/a

Note that full information on the approval of the study protocol must also be provided in the manuscript.

Field-specific reporting

Please select the one below that is the best fit for your research. If you are not sure, read the appropriate sections before making your selection.

[x] Life sciences [ ] Behavioural & social sciences [ ] Ecological, evolutionary & environmental sciences

For a reference copy of the document with all sections, see nature.com/documents/nr-reporting-summary-flat.pdf

Life sciences study design

All studies must disclose on these points even when the disclosure is negative.

Sample size	Power calculations based on existing data from subcutaneous tumours in Kaede murine hosts were used to estimate group sizes for in vivo experiments (PMID: 35472220).
Data exclusions	No data was excluded. For scRNA-seq, droplets were filtered for quality, as described under Methods.

Replication	Findings were validated by different methods, and replicated in 2 or more independent experiments, as described in the manuscript.
Randomization	Animals were not randomly allocated. For all experiments, animals were age and sex matched, and co-housed between experiment and control groups.
Blinding	No blinding. Data presented is quantitative and did not rely on qualitative assessments.

Reporting for specific materials, systems and methods

We require information from authors about some types of materials, experimental systems and methods used in many studies. Here, indicate whether each material, system or method listed is relevant to your study. If you are not sure if a list item applies to your research, read the appropriate section before selecting a response.

Materials & experimental systems

n/a	Involved in the study	
☐	☒	Antibodies
☐	☒	Eukaryotic cell lines
☒	☐	Palaeontology and archaeology
☐	☒	Animals and other organisms
☒	☐	Clinical data
☒	☐	Dual use research of concern
☒	☐	Plants

Methods

n/a	Involved in the study	
☒	☐	ChIP-seq
☐	☒	Flow cytometry
☒	☐	MRI-based neuroimaging

Antibodies

Antibodies used

Target Fluorophore Clone Supplier Catalogue no. Dilution Application
CCR7 AF647 EPR23192-57 Abcam ab275165 1/100 Microscopy
MHC Class II I-A/I-E Pacific Blue M5/114.15.2 BioLegend 107620 1/50 Microscopy
CD3 AF488 / AF647 17A2 BioLegend 100209 / 100210 1/100 Microscopy
CD8a PE 53-6.7 BioLegend 100707 1/50 Microscopy
CD31 AF594 MEC13.3 BioLegend 102520 1/100 Microscopy
4-1BB - AF6-120.1 R&D Systems AF937 1/200 Microscopy
Ki-67 PE SolA15 ThermoFisher 12-5698-82 1/50 Microscopy
Donkey anti-goat AF647 Polyclonal ThermoFisher A32849 1/200 Microscopy
CD16/32 - 2.4G2 BioLegend 101302 1/100 Flow
LIVE/DEAD™ Viability dye APC-Cy7 / NIR - ThermoFisher L10119 / L34981 1/500 Flow
Viability dye VK808 - Beckman Coulter C36628 1/250 Flow
CellTrace Violet - - ThermoFisher C34571 1/1000 Flow
CD45 BV785 / BUV395 30-F11 BioLegend / BD 103149 / 564279 1/200 Flow
CD11c AF647 / AF700 / BV650 N418 Invitrogen 56-0114-82 / 416-0114-82 1/200 Flow
CD11b PE-594 / BV785 / FITC M1/70 BioLegend 101255 / 101243 / 101205 1/200 Flow
XCR1 AF647 / BV510 ZET BioLegend 148213 / 148218 1/200 Flow
Ly6C BV421 / BV711 HK1.4 BioLegend / BD 128031 / 755195 1/250 Flow
MHC Class II I-A/I-E Pacific Blue / BV510 M5/114.15.2 BioLegend 107620 / 107636 1/200 Flow
CCR7 PE-Cy7 / PE 4B12 Invitrogen 12-1971-82 1/100 Flow
PD-L1 BV711 / BV605 10F.9G2 BioLegend 124319 / 124321 1/100 Flow
PD-L2/CD273 BUV395 TY25 BD Horizon 565102 1/200 Flow
CD40 FITC HM40-3 ThermoFisher 11-0402-82 1/100 Flow
CD155 (PVR) PE-Cy7 TX56 BioLegend 131511 1/100 Flow
F4/80 BV605 BM8 BioLegend 123133 1/100 Flow
Ly6G BV650 1A8 BioLegend 127641 1/200 Flow
CD3 BV650 / FITC 17A2 BioLegend 100229 / 100203 1/100 Flow
CD3e FITC / BV605 145-2C11 BD / BioLegend 100305 / 100351 1/100 Flow
CD8a BV510 / BV711 53-6.7 BioLegend 100751 / 100747 1/200 Flow
CD8b APC-Cy7 YTS156.7.7 BioLegend 126619 1/200 Flow
B220 BV650 RA3-6B2 BioLegend 103241 1/200 Flow
NK1.1 BV650 PK136 BD 564143 1/200 Flow
PD-1 BV421 / APC / BV605 29F.1A12 BioLegend 135217 / 135209 / 135219 1/250 Flow
Ki-67 PE-Cy7 SolA15 eBioscience 25-5698-82 1/200 Flow
Granzyme B AF700 QA16A02 BioLegend 372222 1/200 Flow
IFNγ BUV737 XMG1.2 BD 612769 1/200 Flow
CD62L PE-Cy7 MEL-14 BioLegend 104418 1/200 Flow
CD44 PE IM7 ThermoFisher 12-0441-82 1/400 Flow
CD25 PerCP-Cy5.5 PC61.5 Invitrogen 45-0251-82 1/200 Flow

Human CD4 APC RPA-T4 BioLegend 300514 1/100 Flow
Human CD4 BV711 OKT4 BioLegend 317439 1/100 Flow
CD45 BV785 / BUV395 30-F11 BioLegend 103149 / 564279 1/200 FACS
CD11b eFluor 450 / BV785 M1/70 Invitrogen / BioLegend 48-0112-82 / 101243 1/200 FACS
Ter119 PE-Cy7 TER-119 Invitrogen 25-5921-82 1/250 FACS
LIVE/DEAD™ Viability dye APC-Cy7 - ThermoFisher L10119 1/500 FACS
NK1.1 BV650 PK136 BD 564143 1/200 FACS
B220 BV421 RA3-6B2 BioLegend 103239 1/200 FACS
CD11c AF700 / PE N418 Invitrogen 56-0114-82 / 12-0114-82 1/200 FACS
CD3e FITC 145-2C11 BioLegend 100305 1/100 FACS
CD8b APC-Cy7 YTS156.7.7 BioLegend 126619 1/200 FACS
CD62L PE-Cy7 MEL-14 BioLegend 104418 1/200 FACS
CD44 PE IM7 ThermoFisher 12-0441-82 1/400 FACS
MHC Class II I-A/I-E Pacific Blue / BV510 M5/114.15.2 BioLegend 107620 / 107636 1/200 FACS
PD-L2/CD273 BUV395 TY25 BD Horizon 565102 1/200 FACS

Validation

All antibodies used are commercially available. Validation data and antibody data sheets are available on the supplier website using the catalogue number as a reference (provided above). Specificity has been validated by staining cell-lines over-expressing the target antigen or primary immune cells (flow cytometry, immunohistochemistry or western blotting), and compared to isotype controls, and have been used extensively in numerous other studies.

Eukaryotic cell lines

Policy information about cell lines and Sex and Gender in Research

Cell line source(s)

MC38 murine colon adenocarcinoma cells (kindly provided by Dr. Gregory Sonnenberg; Weill Cornell Medicine, New York, NY); sex of cell-line: female.
CT26 murine colon adenocarcinoma cells (kindly provided by Professor Tim Elliot, University of Oxford, Oxford, UK); sex of cell-line: female.
MC38-Ova murine colon adenocarcinoma cells (MC38 expressing ovalbumin, obtained from AstraZeneca); sex of cell-line: female.

Authentication

Cell lines used were not authenticated following procurement.

Mycoplasma contamination

Negative for Mycoplasma contamination.

Commonly misidentified lines
(See ICLAC register)

No commonly misidentified cell lines were used in the study.

Animals and other research organisms

Policy information about studies involving animals; ARRIVE guidelines recommended for reporting animal research, and Sex and Gender in Research

Laboratory animals

Transgenic C57BL/6 Kaede, BALB/c Kaede, OX40L+/Human-CD4 reporter, OX40Lfl/fl, and CD11ccre OX40Lfl/fl mice are maintained and bred at the University of Birmingham Biomedical Services Unit. Wild-type C57BL/6 mice were maintained and bred at the University of Birmingham Biomedical Services Unit or the University of Cambridge Biomedical Services Gurdon Institute animal facilities. Mice were culled between the ages of 8 and 14 weeks. All animal experiments were conducted in accordance with Home Office guidelines and were approved by the University of Birmingham Animal Welfare and Ethical Review Body or the University of Cambridge Animal Welfare and Ethics Review Board. Mice were housed at 21°C, 55% humidity, with 12 h light-dark cycles in 7-7 individually ventilated caging with environmental enrichment of plastic houses plus paper bedding.

Wild animals

No wild animals were used in the study.

Reporting on sex

Both female and male animals were used. Mice were sex-matched for experiments.

Field-collected samples

No field-collected samples were used in the study.

Ethics oversight

All animal experiments were conducted in accordance with Home Office guidelines and were approved by the University of Birmingham Animal Welfare and Ethical Review Body or the University of Cambridge Animal Welfare and Ethics Review Board.

Note that full information on the approval of the study protocol must also be provided in the manuscript.

Plants

Seed stocks: No plants were used in the study.

Novel plant genotypes: n/a

Authentication: n/a

Flow Cytometry

Plots

Confirm that:

- [x] The axis labels state the marker and fluorochrome used (e.g. CD4-FITC).
- [x] The axis scales are clearly visible. Include numbers along axes only for bottom left plot of group (a 'group' is an analysis of identical markers).
- [x] All plots are contour plots with outliers or pseudocolor plots.
- [x] A numerical value for number of cells or percentage (with statistics) is provided.

Methodology

Sample preparation: Tumours were cut into small pieces using surgical scissors, and incubated with 1 mg/ml collagenase D (Roche) and 0.1 mg/ml DNase I (Roche) in a volume of 1.2 ml RPMI media at 37°C on a thermomixer (Eppendorf) for 20 min; or tumours were digested using Tumour Dissociation Kit (Miltenyi Biotec) and gentleMACS Dissociator (Miltenyi Biotec) for 40 minutes at 37°C according to the manufacturer's protocol. The gentleMACS protocol was used for scRNA-seq experiments. Subsequently, the sample was filtered through a 70 µm strainer to remove undigested tissue debris. Next, dead cells were removed using Dead Cell Removal Kit and LS Columns (Miltenyi Biotec), according to the manufacturer's instructions. Lymph nodes were cleaned and dissected in RPMI 1640 medium (Thermo Fisher Scientific) and crushed through a 70 µm strainer. Thereafter, cells were centrifuged at 400 g at 4°C for 5 min and resuspended in FACS staining buffer (2% FBS; 2mM EDTA in PBS) for flow cytometry.
Cell suspensions were subjected to Fc block with anti-CD16/32 (BioLegend) diluted in FACS staining buffer on ice for 15 min before staining with a Live/Dead stain and surface markers, listed in Supplementary table 1, diluted in FACS staining buffer on ice for 30 min. Where applicable, cells were then fixed with Cytofix fixation buffer (BD) for 45 min and stained for intracellular markers diluted in eBioscience permeabilization buffer (Thermo Fisher) at room temperature overnight. 1 x 10^4 counting beads (Spherotech) were added to stained samples at the final step, to calculate absolute cell numbers.

Instrument: Flow cytometry data was collected on the LSR Fortessa X-20 (BD) using FACSDiva v8.0.2 software (BD) or CytoFLEX (Beckman Coulter) using CytExpert v2.5 (Beckman Coulter).

Software: BD FlowJo was used for all analysis, version 10.8.1.

Cell population abundance: Post sort analysis was performed on sorted samples to confirm that the total population was above 99% of total events.

Gating strategy: For FACS: Cell suspensions were stained for CD45 BV786, TER119 PE-Cy7, CD11b BV421, NK1.1 BV650, Live/dead APC-Cy7 on ice for 30 min. Subsequently, cells were centrifuged at 400 g at 4°C for 5 min and resuspended in FACS staining buffer for sorting. Tumour-infiltrating lymphocytes (TIL; Live CD45+TER119-Kaede+CD11b-/lowNK1.1low/hi) and tumour-infiltrating myeloid cells (Live CD45+TER119-Kaede+CD11b+NK1.1-) from tumours were sorted with a FACS Aria II Cell Sorter (BD) into two groups per cell type, based on the presence or absence of Kaede-red signal. CD45+ cells were only sorted to 'myeloid' or 'TIL' (CD11b+ or CD11b-/low respectively) fractions to ensure appropriate representation of various cell types in the scRNA-seq data. All single cell transcriptomes were combined at the analysis stage, before cell type annotation, to ensure all CD45+ immune cells, regardless of surface CD11b or NK1.1 expression, are represented in the final analysis and annotated based on their transcriptome
For flow cytometry: Cells were gated using SSC-A/FSC-A. Singlets were gated using FSC-W/FSC-A. Dead cells were removed with a viability stain, and CD45+ cells were gated. NK1.1- B220- Ly6G- F/480- CD3- Kaede+ MHC-II+CD11c+ cells were gated, to obtain a putative DC population. Activated DCs were gated as PD-L2+CCR7+ cells, because PD-L2 expression was more specific to activated DCs than PD-L1 expression. cDC1 were defined as PD-L2-CCR7-XCR1+ cells, and cDC2 were defined as PD-L2-CCR7-XCR1-CD11b+ cells. For identification of activated CD8 T cells in DC:OT-I co-cultures, singlets (as above) live CD3 +CD8+ CD44+CD62L- cells were gated.

- [x] Tick this box to confirm that a figure exemplifying the gating strategy is provided in the Supplementary Information.
